# Malaria epidemiology in central Myanmar: identification of a multi-species asymptomatic reservoir of infection

**DOI:** 10.1186/s12936-016-1651-5

**Published:** 2017-01-05

**Authors:** Isaac Ghinai, Jackie Cook, Teddy Tun Win Hla, Hein Myat Thu Htet, Tom Hall, Inke ND Lubis, Rosanna Ghinai, Therese Hesketh, Ye Naung, Mya Mya Lwin, Tint Swe Latt, David L. Heymann, Colin J. Sutherland, Chris Drakeley, Nigel Field

**Affiliations:** 1 Research Department of Infection and Population Health, University College London, London, WC1E 6JB UK; 2 Malaria Centre, London School of Hygiene and Tropical Medicine, London, UK; 3 Institute for Global Health, University College London, London, UK; 4 Department of Immunology & Infection, London School of Hygiene and Tropical Medicine, London, UK; 5 Haematology, Severn Deanery, Bristol, UK; 6 University of Medicine (2), Yangon, Myanmar; 7 Department of Infectious Disease Epidemiology, London School of Hygiene and Tropical Medicine, London, UK

**Keywords:** Malaria, Myanmar, Prevalence, Serology, Transmission, Artemisinin, Resistance, Risk factors, Elimination

## Abstract

**Background:**

The spread of artemisinin-resistant *Plasmodium falciparum* is a global health concern. Myanmar stands at the frontier of artemisinin-resistant *P. falciparum.* Myanmar also has the highest reported malaria burden in Southeast Asia; it is integral in the World Health Organization’s plan to eliminate malaria in Southeast Asia, yet few epidemiological data exist for the general population in Myanmar.

**Methods:**

This cross-sectional, probability household survey was conducted in Phyu township, Bago Region (central Myanmar), during the wet season of 2013. Interviewers collected clinical and behavioural data, recorded tympanic temperature and obtained dried blood spots for malaria PCR and serology. *Plasmodium falciparum* positive samples were tested for genetic mutations in the K13 region that may confer artemisinin resistance. Estimated type-specific malaria PCR prevalence and seroprevalence were calculated, with regression analysis to identify risk factors for seropositivity to *P. falciparum*. Data were weighted to account for unequal selection probabilities.

**Results:**

1638 participants were sampled (500 households). Weighted PCR prevalence was low (n = 41, 2.5%) and most cases were afebrile (93%). *Plasmodium falciparum* was the most common species (n = 19. 1.1%) and five (26%) *P. falciparum* samples harboured K13 mutations. *Plasmodium knowlesi* was detected in 1.0% (n = 16) and *Plasmodium vivax* was detected in 0.4% (n = 7). Seroprevalence was 9.4% for *P. falciparum* and 3.1% for *P. vivax*. Seroconversion to *P. falciparum* was 0.003/year in the whole population, but 16-fold higher in men over 23 years old (LR test p = 0.016).

**Discussion:**

This is the first population-based seroprevalence study from central Myanmar. Low overall prevalence was discovered. However, these data suggest endemic transmission continues, probably associated with behavioural risk factors amongst working-age men. Genetic mutations associated with *P. falciparum* artemisinin resistance, the presence of *P. knowlesi* and discrete demographic risk groups present opportunities and challenges for malaria control. Responses targeted to working-age men, capable of detecting sub-clinical infections, and considering all species will facilitate malaria elimination in this setting.

## Background

The last decade has seen global progress in malaria control [1, 2]. This is threatened by the emergence of *Plasmodium falciparum* resistant to artemisinin, the world’s front-line anti-malarial [3]. So far, *P. falciparum* with reduced susceptibility to artemisinin has been described in Cambodia, Thailand, Vietnam, Laos and Myanmar [4–7]. The World Health Organization (WHO) recently set its sights on malaria elimination in Southeast Asia in order to contain this threat [2]. The emergence of the zoonotic primate malaria *Plasmodium knowlesi* as a parasite of significant public health importance in Malaysian Borneo complicates elimination [8, 9]; the distribution and prevalence of this species in other countries in the region remains uncertain.

Myanmar is reported to account for the vast majority of malaria cases and deaths within Southeast Asia [10]. Better understanding the epidemiology of malaria here would likely contribute to successful elimination. Malaria distribution in Myanmar is heterogeneous; forests are a major environmental factor driving patterns of disease [11, 12] and international and internal migration are also risk factors [13, 14]. Whilst transmission is relatively well documented in border areas [13], few data exist from central Myanmar and the burden and drivers of infection in this area remain poorly understood.

Light microscopy and rapid diagnostic tests (RDTs) underestimate the prevalence of infection with *Plasmodium* spp, particularly in low-endemic settings [15]. Polymerase chain reaction (PCR) is more sensitive in detecting current infection and allows further testing such as for drug resistance-associated mutations [16]. Serological methods have been shown to reliably estimate malaria transmission and can identify risk factors with achievable sample sizes in low prevalence areas [17]. Prevalence of antibodies to specific malaria antigens, which indicate malaria exposure, can be used to calculate the seroconversion rate (SCR, equivalent to the force of infection) [18]. Changes in SCR over time are of particular utility in monitoring the success of public health campaigns.

This study combined highly sensitive PCR testing and serological evidence of transmission intensity in a household survey in central Myanmar with collection of detailed epidemiological data to investigate the prevalence of, and risk factors for, malaria exposure.

## Methods

### Study site and population

**Fig. 1:**
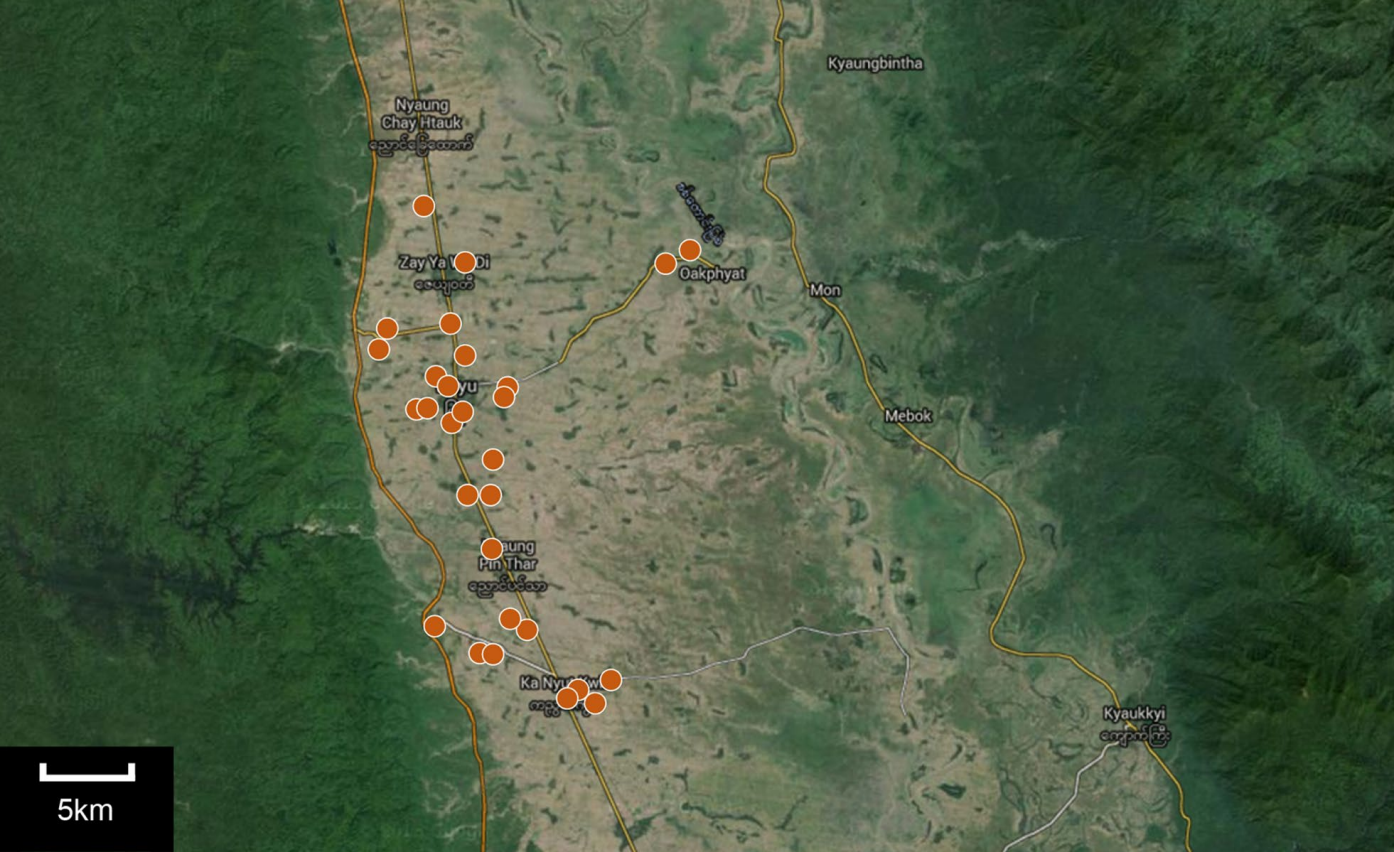
Study site with GPS points (*circles*) demarcating each village cluster

Phyu is the largest township in Eastern Bago Region. Phyu is located on the main highway between Yangon (largest city) and Nay Pyi Taw (capital). It is 100 km west of the border with Thailand, and lies on a plain (highest altitude = 60 m) between forest-covered mountains (Fig. 1). The forests attract migrant labour for logging, charcoal production and agro-forestry [14]. Phyu was selected for this study due to its relatively large size and location on busy transport routes near forests. The population is approximately 250,000, of whom 89% live in semi-urban or rural areas, with 11% in Phyu town.

Medical services vary from a 50-bed township hospital to sub-rural health centers with no inpatient facilities. The latter cover 5–15 villages each with a midwife who provides basic services including malaria prevention. The township malaria inspector and local midwives accompanied the team for this study.

The monsoon begins in May and ends in October; precipitation is heaviest in August (approximately 450 mm/month). Peaks in malaria transmission follow the rains by 1–2 months. This study was conducted in August and September 2013.

### Field procedures

#### Sample size calculation

A required sample size of 1174 was calculated, assuming a malaria prevalence of 1 per 1000, with 95% confidence with 80% power; sampling 0.1% of the population, with a presumed design effect of 2 and a non-response rate of 15%. Since the average household size in Myanmar is 4.7, 30 clusters of 20 houses (~75 participants) were chosen (n = 1500).

#### Sampling technique

This cross-sectional study used a multistage cluster random sample design. Administrative wards were the primary sampling unit, with villages and houses as the secondary and tertiary sampling units. 26 of 61 wards were included (population = 152,651, 60.2% of the total). The remaining wards (population = 101,078, 39.8%) were defined as inaccessible by vehicle by the local government, necessitating exclusion. The included wards were weighted according to population size and randomly selected.

From each selected ward, villages were selected at random with no population weighting. If a ward was selected more than once, more than one village was selected. Each selected village represented one cluster. A map was drawn by village leaders and houses were selected by systematic interval sampling and mapped using a Global Positioning System. Houses with no response were not revisited, the house with the closest street entrance was sampled in its place.

### Questionnaires

Questionnaires were adapted from publicly available malaria indicator survey toolkits [19]. Pre-tested questionnaires were administered by pairs of community healthcare workers trained for this study. Households were defined as the group of people who had slept under the same roof the previous night and who shared a common entrance to the street. Household questionnaires recorded household structure, assets and demographic information for every household member, including those absent. Individual questionnaires recorded self-reported forest exposure, malaria symptoms and malaria prevention practices.

Participants were categorized as having exposure to forest if their occupation involved forest-related work (forestry, farming, labouring) or if they reported visiting the forest within the previous 6 months. Households were classified as having universal coverage of bed nets if they reported having at least 1 net per 2 people. Socio-economic status (SES) was calculated using principal components analysis including household structure and assets. The derived score was divided into tertiles.

### Clinical samples

Tympanic temperature was measured for all participants. Febrile participants (≥37.5 °C) were tested using RDT (SD Bioline Malaria Ag Pf/Pan) and referred for treatment. Capillary blood samples were spotted directly onto Whatman 3MM filter paper for all participants over 6 months old regardless of tympanic temperature. Blood spots were air-dried, sealed with silica gel and sent to the London School for Hygiene and Tropical Medicine for storage at −20 °C before analysis.

### Ethical approval

The study received approval from the Research Ethics Committee of University College London, the Myanmar Ministry of Health and Department of Medical Research, Lower Myanmar. Local and regional authorities were informed. Informed consent was obtained from all adults and from guardians of children aged under 15. For those aged between 15 and 18, informed consent from both parent and child was obtained.

### Laboratory procedures

#### Serological analysis

Recombinant proteins: *Plasmodium falciparum* and *Plasmodium vivax* antigens MSP-1_19_ were expressed in *Escherichia coli* as glutathione *S*-transferase-fusion proteins [20] (Pv provided by A. Holder). PfAMA1_3D7 and PvAMA1_Sal1 were expressed as His-tagged proteins [21, 22].

Detection of anti-malarial antibodies by ELISA: IgG antibodies to each recombinant protein were assessed by ELISA as previously described [23]. Briefly, recombinant antigens were coated overnight at 4 °C at the following concentrations: PfAMA-1 at 0.5 μg/ml, PfMSP-1_19_ at 0.18 μg/ml, PvAMA-1 at 1 μg/ml and PvMSP-1_19_ at 1.8 μg/ml. Plates were washed using PBS plus 0.05% Tween 20 (PBS/T) and blocked with 1% skimmed milk powder in PBS/T. Samples and positive control sera (a pool of hyperimmune serum collected from a malaria endemic area) were tested in duplicate at 1/1000 and 1/2000 dilutions against MSP and AMA, respectively. After washing, horseradish peroxidase-conjugated polyclonal rabbit anti-human IgG (Dako, Glostrup, Denmark) diluted at 1:5000 in PBS/T was added to all wells. All plates were developed using OPD substrate (OPD, sigma fast) and reactions were stopped with 2 M H_2_SO_4_. Plates were read at 492 nm and optical density (OD) values were recorded. Seropositivity was determined using a finite mixture model to define a cut-off value as previously described [18].

#### PCR analysis

Parasite DNA from blood spots was extracted using the Chelex method [24]. PCR amplifications targeting the 18S rRNA gene was performed for initial screening for *Plasmodium* infections, using published PCR reactions and protocols with minor modifications [8]. PCR-positive samples were subsequently tested using an additional method targeting the cytochrome B gene (*cytb*) designed for the current study. The primer sets for the hemi-nested *cytb* assay were as follows: PgCytbF1 (5′-GAATTATGGAGTGGATGGTG-3′) and PgCytbR1 (5′-ACATCCAATCCATAATAAAGC-3′) for nest 1 and PgCytbF1 and PgCytbR2 (5′-TTTTAACATTGCATAAAATGG-3′) for nest 2. Amplification cycling conditions were 95 °C for 3 min and then 30 cycles of 3 steps PCR, with 30 s annealing at 55 °C (nest 1) or 58 °C (nest 2) and elongation at 72 °C for 1 min. Nested products were then characterized by direct sequencing for species determination. Polymorphisms at the propeller domain of the Kelch 13 (K13) encoded by the *P. falciparum k13* gene (PF3D7_1343700) were also determined on *P. falciparum* PCR-positive isolates, using previously described protocols for nested PCR methods and DNA sequencing [25].

#### Microscopic analysis

Thick and thin blood films were taken from each participant. Giemsa-stained thick malaria films were examined under a 100× oil-immersion lens. Samples were counted as negative if no parasite was seen after counting 400 white blood cells. Samples were counted as positive if one or more parasites were seen—the corresponding thin films were then examined with a 100× oil-immersion lens for speciation. All microscope slides were read in duplicate by technicians blinded to other reader’s findings.

#### Data analysis

Weighted prevalence of malaria species by PCR and serological status was estimated with 95% confidence intervals using the Stata survey commands (v14, Statacorp, Texas).

To investigate transmission dynamics in the population, a simple reversible catalytic conversion model was fitted to the age-seroprevalence data using maximum likelihood methods as previously described [17]. The model fits age-seroprevalence curves and estimates seroconversion rate (SCR) as analogous to the force of infection. Profile likelihood plots were used to test for any step change in SCR and, if present, identify the most likely age at which this occurred [26]. Models with two SCRs instead of one were assumed if the likelihood ratio comparing models indicated a statistically significant change (p < 0.05).

Univariate and multivariate logistic regression was used to investigate the relationship between gender, age, ethnicity, bed net ownership, SES, forest exposure, and seropositivity to *P. falciparum* antigens.

## Results

A total of 701 houses in 27 clusters (villages) across 14 administrative wards were visited, of which 500 houses had at least one resident present. Of the 2112 recorded residents of consenting households, 474 (22.4%) were absent (Table 1). 1638 were present and all consented to take part in the study.

**Table 1:** Demographic characteristics of study participants

	Study population	
	n	%
Total	1638	100
Gender		
Males	752	45.9
Female	886	54.1
Age		
0–5	163	9.9
6–15	324	19.8
16–25	255	15.6
26–50	550	33.6
51–100	346	21.1
Ethnicity		
Burmese	1086	64.1
Indian	507	33.0
Shan	45	2.9
Socioeconomic status		
Lowest	513	32.4
Middle	549	34.6
Highest	523	33.0
Work in or visit forest		
Yes	671	41.0
No	967	59.0
Household bed net ownership		
Yes, universal coverage	398	79.6
No	102	20.4

The median age was 30 years (range 6 months-88 years) and 55% were female. 43% (57% of men and 27% of women) were classified as having had forest exposure (Table 1). 80% of households were classified as having universal bed net coverage, but very few households were reported as having been sprayed with insecticide in the past year (3%). The majority of participants were of Burmese origin (63%) whilst people of Indian origin represented 33% of the sample and Shan origin 3%. Absent household residents were more likely to be male and less likely to be Indian compared to those included in the study.

### Malaria indicators

**Fig. 2:**
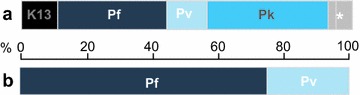
Proportion of *Plasmodium* species identified in **a** our sample and **b** WHO reporting for Myanmar. Pf, *P. falciparum*; Pv, *P. vivax*; Pk, *P. knowlesi*. K13 Pf samples with K13 mutations (not available for WHO statistics). * 1 × Pf Pv, 1 × Pk Pv, 1 × *P. malariae*

41 participants were found to have current infection when tested post hoc by PCR (2.6%, CI 1.5–4.3) (Table 2). The most common species detected was *P. falciparum* (1.1%, n = 19), followed by *P. knowlesi* (1.0%, n = 16). *Plasmodium vivax* (0.5%, n = 7) and *Plasmodium malariae* (0.08%, n = 1) (Fig. 2). Only two mixed infections were detected. Three infected individuals were febrile (one each of *P. falciparum, P. knowlesi* and *P. vivax*), the remaining 38 were afebrile (93%). No infected individuals reported any other malaria symptoms and none had sought treatment for malaria in the past three months. 89 febrile people were tested with an RDT and none were found positive, including three PCR-confirmed infections. *P. falciparum* and *P. knowlesi* infections were found in all age groups. *Plasmodium knowlesi* was most common between ages 5 and 15. *Plasmodium vivax* was only detected in those aged 15 and above.

**Table 2:** Prevalence of symptoms (fever) and specific *Plasmodium* species by PCR and serology

	n	Prevalence (95% CIs)	Proportion of current infection (%)
Fever	89	4.9 (3.5–7.1)	3/89 (3.4%)
PCR			
Any *Plasmodium* species	41	2.6 (1.5–4.3)	100^a^
*P. falciparum*	19	(0.4–3.0)	46.3 (28.7–59.1)
*P. knowlesi*	16	(0.5–2.2)	39.0 (21.9–51.3)
*P. vivax*	7	0.5 (0.1–2.0)	17.1 (2.2–22.2)
*P. malariae*	1	0.04 (0.0–0.4)	2.4 (0–7.08)
Seropositive			
*P. falciparum*	150	9.4 (6.0–15.0)	
*P. vivax*	50	3.1 (1.9–6.3)	

^a^Includes two mixed infections, one *P. falciparum* and *P.vivax,* one *P. knowlesi* and *P. vivax*, therefore total percentages may not equal 100%

5 of the 19 (26.3%) *P. falciparum* samples harboured non-synonymous propeller domain mutations in *pfk13* (K13). These included the known variant Val534Ala [27] and four novel variants: Arg539Val, Asn572Asp, Glu602Gly, and Glu620Gly.

9.4% (n = 150) individuals were seropositive to *P. falciparum* antigens whilst 3.1% (n = 50) were seropositive to *P. vivax* antigens. Of the 50, 19 (38%) were also positive to *P. falciparum*, indicating exposure to both species. Seropositivity to both *P. falciparum* and *P. vivax* was higher in men than women for both species (p value = 0.018 and 0.063 respectively).

Microscope slide positivity had high inter-observer variability, in part due to water damage and fungal contamination of slides, and the microscopy results were therefore not included in the analysis.

### Risk factors for exposure to malaria

Analysis of risk factors for malaria was restricted to seropositivity to *P. falciparum* due to the low numbers of infections detected by PCR. After adjusting for all other variables, seropositivity was more likely for men, [adjusted OR (AOR) 2.1 (1.3–3.4), p = 0.006], and less likely for those with higher SES [AOR 0.6 (0.3–0.9), p = 0.072], and of Indian origin [AOR 0.3 (0.1–0.8)]. There was a strong association between seropositivity and forest exposure in the unadjusted model, which was lost after adjusting for gender, age, ethnicity and SES (Table 3). Migration status (moving to Phyu within the last 10 years or visiting outside Phyu in the preceding 3 months) was not associated with an increased risk of malaria exposure in either the crude or adjusted analysis.

**Table 3:** Association between demographic and behavioural characteristics and exposure to *P. falciparum* in study participants

	Sero-positive (%)	Odds ratio (OR)	p value	Adjusted OR^a^	p value	Denominators	
						Un-weighted	Weighted
Gender							
Female	7.1	1.0 (baseline)		1.0		862	871
Male	12.6	1.9 (1.3–2.8)	0.004	2.1 (1.3–3.4)	0.006	740	731
Age							
0–5 years	0.3	1.0		1.0		162	181
5–15 years	3.1	9.0 (1.4–58.9)		9.0 (1.3–64.8)		311	292
15–25 years	3.8	11.2 (1.6–81.5)		12.0 (1.5–94.8)		253	265
25–50 years	13.0	42.7 (4.0–456.3)		43.8 (3.8–506.3)		541	544
50–100 years	20.0	71.2 (6.4–796.3)	0.014	71.3 (5.6–904.2)	0.030	335	319
Working in/visiting forests							
No	5.8	1.0		1.0		1454	1454
Yes	14.7	2.8 (1.5–5.1)	0.002	1.0 (0.6–1.8)	0.979	31	31
Bed net ownership							
Not universal coverage	6.6	1.0		1.0		391	417
Universal coverage	11.0	1.8 (0.8–3.8)	0.134	1.1 (0.5–2.5)	0.760	1161	1145
Socio-economic status							
Low	11.6	1.0		1.0		506	462
Medium	11.2	0.9 (0.6–1.5)		0.9 (0.6–1.6)		532	531
Highest	7.2	0.6 (0.4–0.9)	0.035	0.6 (0.3–0.9)	0.072	512	566

^a^Adjusted for: age, gender, forest exposure, ethnicity, bed net ownership and socio-economic status

### Serological estimates of malaria transmission

**Fig. 3:**
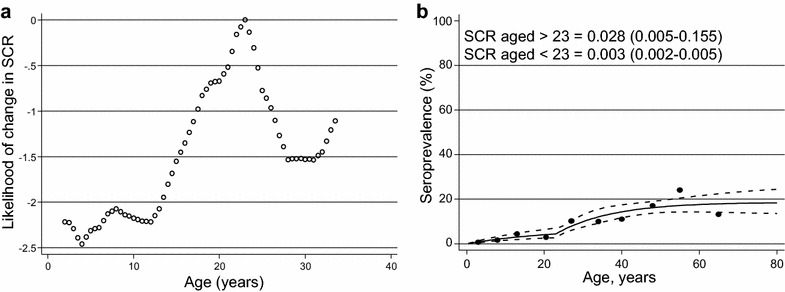
**a** Profile likelihood plot investigating the most likely age at which a change in seroconversion rate (SCR) occurs in *P. falciparum* serological data and **b** Seroprevalence curve fitted to *P. falciparum* with a change in SCR at age 23

A reversible catalytic model and profile likelihood plots were used to investigate whether there was evidence for two SCRs, indicative of two discrete levels of exposure within the population. For *P. falciparum*, there was a clear peak in profile likelihood plot at age 23 years, indicating a change in SCR (Fig. 3a). A model allowing for two SCRs fitted the data significantly better than a model with a single SCR (p = 0.016) (Fig. 3b).

**Fig. 4:**
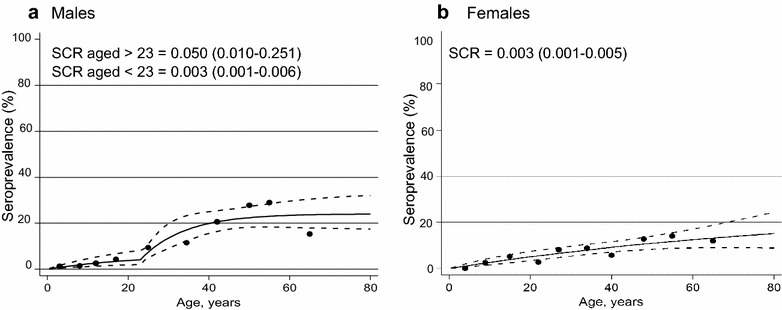
Seroprevalence curves for *P. falciparum* **a** males and **b** females. The data for males has been fitted with two serconversion rates (SCR), with a change at age 23, and the data for females has been fitted with a constant SCR

When analysed separately by gender, a model with two SCRs fitted better for men (p = 0.004) whilst a single SCR fit better for women (p = 0.548) (Fig. 4). The SCR in men over the age of 23 was 0.050 (0.010–0.251) compared to 0.003 (0.001–0.006) in those under 23 years old. In women, the SCR was similar to that of men under 23 [0.003 (0.001–0.005)].

Seropositivity to *P. vivax* antigens was low and SCR was also low [0.002 (0.0009–0.006)], with no evidence for a change in SCR.

## Discussion

This household study reveals low-level malaria transmission among the general population in central Myanmar. The WHO estimates that *P. falciparum* causes 75% of clinical malaria in Myanmar and *P. vivax* 25% [28], whilst in this study *P. falciparum* mono-infection comprised 45% of infections and *P. vivax* 12% (Fig. 2). This study found *P. knowlesi* [29] in more than 35% of positive samples. Clinical diagnosis of malaria and most epidemiological studies in Myanmar use microscopy or RDT positivity to identify infection and both RDT and microscopy have poor sensitivity and specificity for *P. knowlesi* [30, 31]. In this study, reliable microscopy also proved technically challenging. This may explain the underrepresentation of *P. knowlesi* in national statistics and population-level data when compared to the findings presented here. In Malaysia, *P. knowlesi* is responsible for a similar proportion of cases as found in this study and can cause fatal illness [32]. The epidemiology of *P. knowlesi* in Myanmar requires further study, utilizing sensitive diagnostic tools.

One quarter of *P. falciparum* samples harboured K13 mutations, all from afebrile, asymptomatic participants, none of whom reported anti-malarial treatment within the previous three months and none were followed up—it is therefore difficult to comment on the importance of these alleles as determinants of artemisinin susceptibility. Of the five K13 mutations detected, four have never previously been reported. One of the novel mutations, Arg539Val, is similar to previously described variants at codon 539 from Cambodia, Laos and Myanmar, though these all encode threonine rather than valine as the variant amino acid [27]. K13 mutations have recently been mapped in convenience samples in Myanmar and appear prevalent in Upper Myanmar and eastern border areas; no K13 mutations were found in a clinical cohort of 52 patients in Bago Region in 2013–2014 [33], although the prevalence of K13 mutations amongst clinical cohorts in the high-risk township of Shwe Kyin, Bago, was similar to that found in our population [5].

Most participants (n = 38; 93%) with current infection were afebrile and none reported symptoms. The three individuals with raised temperatures tested negative on RDT, possibly signifying a low-density parasitaemia (or alternative diagnoses) which may be associated with mild clinical symptoms [34]. However, even sub-clinical infections are important during elimination campaigns [35]; accurate identification and treatment of asymptomatic carriers—who might transmit infections and will tend not to seek treatment—proved crucial to eliminate malaria in a similar low-endemic setting [36].

The serological data support the PCR data and suggest that there has been ongoing low-level transmission of *P. falciparum* and *P. vivax* in the general population in Bago region. There was a notable increase in exposure to infection in men aged over 23, who experience a SCR 16-fold higher than the general population. Because the antibodies detected by these methods persist for an unknown period of time, there are two explanations indistinguishable by serology alone: malaria epidemiology may have changed 23 years ago such that men now aged over 23 were historically exposed to more malaria, or there may be ongoing behavioural risk factors specific to those aged over 23. The best working hypothesis is that the change in SCR is behavioural; it is confined to a well-defined epidemiological group (men of working age) and this population is overrepresented in contemporary clinic-based studies suggesting they remain at increased risk of malaria [16].

Forest work is an established risk factor for malaria transmission [37]. *Plasmodium knowlesi* is particularly associated with forest malaria, due to the outdoor biting habits of *P. knowlesi* vectors and the natural primate reservoirs living in forests [38]. The questionnaire captured self-reported information relating to occupational exposure and forest visits. Individuals reporting forest exposure were three times more likely to have evidence of previous exposure to malaria in the unadjusted analysis. However, forest workers were significantly more likely to be men and aged over 23 and less likely to be Indian. After adjusting for other factors including age, ethnicity and SES the association with forest exposure was no longer significant, which is consistent with forest exposure being on the causal pathway (although it cannot be excluded that it is a confounder). Illicit forest-related activities such as illegal logging are often unreliably reported in surveys, and qualitative studies may be needed to obtain more information on the spectrum of forest exposure as a risk behaviour for malaria in this region.

The Myanmar Ministry of Health offers malaria prevention, diagnosis and treatment to the general population without distinction. This approach is reflected in the high level of bed-net ownership and might contribute to the low levels of transmission observed. However, these data suggest that a strategy tailored to those at highest risk and with asymptomatic infection might be required to eliminate malaria since bed-nets will not protect those working in forest plantations at dawn and dusk [14]. Targeted interventions have been trialed in other areas with varying degrees of success, including: insecticide-treated clothing and hammocks (for use in the forest); toxic mosquito baits; and personal insect-repellents [39]. Targeted mass administration of anti-malarials to specific demographic groups is another option [40]. As yet, none have been evaluated in Myanmar.

This is the first study in Myanmar to combine detailed population-level epidemiological data with highly sensitive molecular techniques. The strength and novelty of this study also lie in the size of this probability sample from an important but unstudied population, with generalizability to Bago Region and central Myanmar. However, higher diagnostic sensitivity has been achieved with the technique of high volume ultra-sensitive qPCR [16] though this requires collection of larger blood volumes by venepuncture, and an unbroken cold-chain for transport of packed red cells to a specialist molecular biology laboratory. Point prevalence of current infection is sensitive to seasonal variation, and this study was conducted towards the end of the rains—regarded as the malaria high season—to sample at the peak of malaria transmission (though different seasonal peaks have been reported for some areas nearby [41, 42]). Moreover, others report little by way of seasonal variation [43], and by correlating PCR findings with serology, which is less sensitive to seasonal changes, robust hypotheses can be developed. The study aimed to minimize bias in this population-based study by weighting the sample so that it was broadly representative of national demographic data in Myanmar in terms of gender and age [44], but the sampling strategy excluded the population living closest to the flooded Sittang River due to adverse weather conditions, which made these areas inaccessible, and where malaria transmission might be higher. In addition, the ethnic composition of this sample differs from national averages because ethnicity in Myanmar is clustered [44]; the sample over-represented Indian participants (33% compared to 2% in national statistics) and underrepresented Shan (3% compared to 9%). One-fifth of residents were absent during study visits, and these individuals were more likely to be male and less likely to be of Indian origin, and this household survey did not include hospital in-patients being treated for malaria. We were only able to provide population weighting at the ward level due to a lack of available population statistics for individual villages. In our experience, village sizes did vary, though it is unlike that this would have affected our prevalence estimates, given the ward-level weighting applied and the small number of infections detected. The overall effect of our diagnostic techniques and sampling strategy is that the study design might have underestimated rather than overestimated malaria prevalence.

## Conclusion

Malaria transmission in this area was low. However, there were low numbers of asymptomatic carriers who probably play an important role in maintaining local transmission, and many were infected with *P. knowlesi*. A higher proportion of K13 mutations among *P. falciparum* cases were observed than has previously documented in this region, and working age men appear at significantly higher risk of malaria exposure when compared to the general population, which needs further research. However, the public health implications are clear; the WHO goal of malaria elimination in this region will require identification and treatment of asymptomatic carriers among high-risk populations in order to control artemisinin resistance and successfully eliminate malaria in this setting.

## Abbreviations

PCR: polymerase chain reaction
K13: propeller region of the kelch protein on chromosome 13 of the *Plasmodium falciparum* genome (mutations here may be responsible for conferring artemisinin resistance)
RDT: rapid diagnostic test for malaria
WHO: World Health Organization
SCR: seroconversion rate (the rate at which individuals develop antibodies to malaria antigens)
